# Supplementary material for: 2D-QSAR-guided design of potent carbamate-based inhibitors of acetylcholinesterase
Source: PLoS One. 2025 May 20;20(5):e0320789. doi: 10.1371/journal.pone.0320789 (PMC12092016; doi:10.1371/journal.pone.0320789)
Supplement: Table S1 — (DOCX) [file pone.0320789.s001.docx]

**Design of novel potent inhibitor based on 2D-QSAR of carbamate derivatives for AChE inhibition**

Meriem Khedraoui ^(1)^, El Mehdi Karim ^(1)^, Oussama Abchir ^(1)^, Abdelkbir Errougui ^(1)^,
Yasir S. Raouf ^(2)^, Abdelouahid Samadi ^(2)^, Samir Chtita ^(1, *)^

^(1)^ Laboratory of Analytical and Molecular Chemistry, Faculty of Sciences Ben M’Sik, Hassan II University of Casablanca, Casablanca, Morocco

^(2)^ Department of Chemistry, College of Science, United Arab Emirates University, Al Ain P.O. Box 15551, United Arab Emirates;

* Corresponding authors: A.S. [samadi@uaeu.ac.ae](mailto:samadi@uaeu.ac.ae); S.C. [samirchtita@gmail.com](mailto:samirchtita@gmail.com)

**Supplementary material**

Table S1. Structures and pIC_50_ values of the 32 studied compounds

| ****  **pIC_50_ = 6.60** | ****  **pIC_50_ = 6.41** | ****  **pIC_50_ = 6.53** |
| --- | --- | --- |
| **1** | **2** | **3** |
| ****  **pIC_50_ = 6.93** | ****  **pIC_50_ = 6.93** | ****  **pIC_50_ = 7.42** |
| **4** | **5** | **6** |
| ****  **pIC_50_ = 7.38** | ****  **pIC_50_ = 8.08** | ****  **pIC_50_ = 7.59** |
| **7** | **8** | **9** |
| ****  **pIC_50_ = 7.32** | ****  **pIC_50_ = 7.25** | ****  **pIC_50_ = 5.90** |
| **10** | **11** | **12** |
| ****  **pIC_50_ = 6.26** | ****  **pIC_50_ = 6.29** | ****  **pIC_50_ = 6.29** |
| **13** | **14** | **15** |
| ****  **pIC_50_ = 6.42** | ****  **pIC_50_ = 6.56** | ****  **pIC_50_ = 6.53** |
| **16** | **17** | **18** |
| ****  **pIC_50_ = 6.61** | ****  **pIC_50_ = 6.82** | ****  **pIC_50_ = 6.50** |
| **19** | **20** | **21** |
| ****  **pIC_50_ = 6.95** | ****  **pIC_50_ = 6.36** | ****  **pIC_50_ = 6.69** |
| **22** | **23** | **24** |
| ****  **pIC_50_ = 6.81** | ****  **pIC_50_ =7.67** | ****  **pIC_50_ = 6.82** |
| **25** | **26** | **27** |
| ****  **pIC_50_ = 7.52** | ****  **pIC_50_ = 7.64** | **pIC_50_ = 7.19** |
| **28** | **29** | **30** |
| ****  **pIC_50_ = 7.23** | ****  **pIC_50_ = 7.26** |  |
| **31** | **32** |  |
