# Supplementary material for: 2D-QSAR-guided design of potent carbamate-based inhibitors of acetylcholinesterase
Source: PLoS One. 2025 May 20;20(5):e0320789. doi: 10.1371/journal.pone.0320789 (PMC12092016; doi:10.1371/journal.pone.0320789)
Supplement: Table S2 — (DOCX) [file pone.0320789.s002.docx]

**Design of novel potent inhibitor based on 2D-QSAR of carbamate derivatives for AChE inhibition**

Meriem Khedraoui ^(1)^, El Mehdi Karim ^(1)^, Oussama Abchir ^(1)^, Abdelkbir Errougui ^(1)^,
Yasir S. Raouf ^(2)^, Abdelouahid Samadi ^(2)^, Samir Chtita ^(1, *)^

^(1)^ Laboratory of Analytical and Molecular Chemistry, Faculty of Sciences Ben M’Sik, Hassan II University of Casablanca, Casablanca, Morocco

^(2)^ Department of Chemistry, College of Science, United Arab Emirates University, Al Ain P.O. Box 15551, United Arab Emirates;

* Corresponding authors: A.S. [samadi@uaeu.ac.ae](mailto:samadi@uaeu.ac.ae); S.C. [samirchtita@gmail.com](mailto:samirchtita@gmail.com)

**Supplementary material**

Table S2. Structures and predicted pIC_50_ values of new designed compounds

|  | **Structures** | **C.A.E.** | **E_LUMO_** | **Hydrogen %** | **pIC_50_** | **hi** | **Outlier / Inside** | |
| --- | --- | --- | --- | --- | --- | --- | --- | --- |
| **M1** | **** | 857.82 | -0.060 | 7.360 | 8.220 | 1.213 | Outlier |  |
| **M2** | **** | 939.47 | -0.054 | 6.890 | 8.200 | 0.223 | Inside |  |
| **M3** | **** | 924.79 | -0.055 | 7.580 | 8.300 | 0.380 | Inside |  |
| **M4** | **** | 924.36 | -0.054 | 7.580 | 8.290 | 0.300 | Inside |  |
| **M5** | **** | 937.93 | -0.053 | 8.260 | 8.500 | 0.274 | Inside |  |
| **M6** | **** | 938.36 | -0.053 | 9.260 | 8.760 | 0.417 | Inside |  |
| **M7** | **** | 925.51 | 0.053 | 7.310 | 8.220 | 0.205 | Inside |  |
